# Comparison of the histopathology and prognosis of bilateral versus unilateral multifocal multicentric breast cancers

**DOI:** 10.1186/1477-7819-12-266

**Published:** 2014-08-20

**Authors:** Hüseyin Kadioğlu, Serdar Özbaş, Alper Akcan, Aykut Soyder, Lutfi Soylu, Savaş Koçak, N Zafer Cantürk, Mustafa Tükenmez, Mahmut Müslümanoğlu

**Affiliations:** Department of General Surgery, Bezmialem Vakif University, Istanbul Adnan Menderes Bulvarı Vatan Caddesi, 34093 Fatih/İstanbul, Turkey; Department of General Surgery, Güven Hospital, Şimşek Sok. No:29, 06540 Kavaklıdere, Ankara Turkey; Department of General Surgery, Erciyes University, Erciyes Üniversitesi Tıp Fakültesi Dekanlığı, 38039 Talas/Kayseri, Turkey; Department of General Surgery, Adnan Menderes University, 09100 Aydın, Turkey; Department of General Surgery, Kocaeli University, 41380 Umuttepe/KOCAELİ, Turkey; Department of General Surgery, Istanbul University Medical Faculty, 34083 Istanbul, Turkey

**Keywords:** Bilateral synchronous, Multifocal, Multicentric, Breast cancer

## Abstract

**Background:**

Multiple breast cancers may present with different clinical and biological characteristics. The data indicate that multifocal (MF), multicentric (MC), and bilateral synchronous (BS) breast cancers (BC) are more aggressive and have an equivalent or moderately poorer survival rate compared with unilateral cases. However, a comparison of these multiple breast cancers has not been covered in the literature. The aim of this study was to describe the histopathological characteristics of patients suffering from MF, MC, and BS breast carcinoma and to compare their prognoses.

**Methods:**

Retrospective data for MF, MC, and BS breast carcinoma patients treated in five different breast cancer units in Turkey between 2003 and 2012 were collected. MF and MC cancers were defined as more than one lesion in the same quadrant or in separate quadrants, respectively.

**Results:**

There were 507 patients (271 MF, 147 MC, and 89 BS) treated in this time period. BS breast carcinoma patients were younger than the other groups (44.83 ± 9.6, 47.27 ± 11.6, and 51.11 ± 11.8 years for BS, MF, and MC breast carcinoma patients, respectively). MFBC and MCBC patients in this study were younger than the ages reported in Western literature, but this result was similar to the ages reported in Eastern literature. The five-year survival rates and recurrence rates were not statistically different among groups (*P* = 0.996 and *P* = 0.263, respectively). According to univariate analyses, tumor size, histological grade, and lymph node status were statistically significant factors that affected survival. However, only lymph node involvement was significant for survival according to multivariate analyses.

**Conclusions:**

The clinical significance of MF, MC, and BS breast cancers is still unclear and their influence on prognosis is controversial. Disease-free and overall survival rates of BS breast cancers might be similar to MF and MC breast cancers.

## Background

Unilateral, multifocal, and multicentric breast cancers have been of interest to oncology professionals for many years, but their impact on prognosis and survival is controversial [1]. While early clinical trials advocated mastectomy for multifocal and multicentric tumors [2], the recent literature suggests local recurrence rates do not differ from unifocal tumors [3].

Bilateral synchronous breast cancer (BSBC) is a rare entity with an incidence of between 1 and 3%. Surprisingly, there has been no increase in BSBC incidence since the 1980s [4]. However, its incidence has been reported to be as high as 12% [5]. Different time intervals have been used to define BSBC. In 1921, Kilgore defined BSBC as a breast cancer in which both tumors are diagnosed at the same time [6]. Since 1921, different time intervals have been used ranging from one month to five years [7]. A widely accepted definition of BSBC is that of Hartman *et al*., who defined BSBC in 2007 as a tumor diagnosed within 90 days of the first tumor [4].

Another dilemma regarding BSBC is the relationship between tumors. Contradictory results on BSBC have been published. Some authors have demonstrated similarity in histologic subtype [8], tumor grade [9], and hormone receptor status [10] between tumors, suggesting a single-cell origin, but other authors have reported different results [11]. Some authors believe that the tumors are genetically different [12].

The survival effect of BSBC is also a dilemma for oncology professionals. While some reports advocate a worse prognosis for BSBC, other reports do not suggest a worse prognosis for BSBC.

According to the current literature, multifocal breast cancer (MFBC) does not affect survival. Recent studies have compared BSBCs with unilateral breast cancer, unifocal breast cancer, MFBC, and multicentric breast cancer (MCBC). We believe that this heterogeneity is the cause of the differences reported in survival ratios, and that tumors beginning from more than one focus should be compared with other tumors beginning from more than one focus.

The aim of this study was to compare the histopathological findings and survival of BSBC, MFBC, and MCBC cases and to analyze the effect of molecular subtype on survival.

## Methods

The retrospective data for BSBC, MFBC, and MCBC patients treated in five different breast centers in Turkey between 2003 and 2012 were collected.

All of these centers are university hospitals and have a similar follow-up schedule for breast cancer based on national comprehensive cancer network (NCCN) guidelines. The data were obtained from the oncology departments’ follow-up files of the patients.

The following patients were excluded from the study: patients who were treated with neo-adjuvant chemotherapy, patients who had a history of contralateral breast cancer, patients who received chemotherapy or radiotherapy for any kind of malignancy, patients who were male; patients who were lost at follow-up, and patients who were not seen by a medical professional in the previous six months.

Patients diagnosed with contralateral breast cancer in the most recent 90 days were defined as BSBC. MFBC was defined as tumors separated by normal breast tissue in the same quadrant, and MCBC was defined as tumors separated by normal breast tissue in different quadrants. Multifocal and multicentric tumors were identified by macroscopic examination of pathology specimens. *In situ* components of the tumors were not added to the tumor size. Tumors connected by *in situ* cancer were defined as asunder foci. Also tumors which are closer than 5 mm to each other are defined as satellite nodules.

A total of 507 patients met these criteria. Age at diagnosis, menopausal status, number of tumor foci (index side for BSBC), tumor size (index side for BSBC), histological type (index side for BSBC), histological grade (HG; defined using the Modified Bloom-Richardson grading system), lymphovascular invasion (LVI) status, lymph node involvement, estrogen receptor status, progesterone receptor status, HER2/neu status, stage, and molecular subtype were recorded.

All five centers were using ultrasonography for the preoperative evaluation of the axillary, supra-clavicle, and internal mammary lymph nodes. All five centers were using immunohistochemical methods to define HER2/neu status up to year 2009; *in situ* hybridization techniques have been in use since year 2009.

Estrogen receptor-positive patients with low Ki 67 levels (<14%), HER2/neu-negative, and low-grade tumors were defined as luminal A. Estrogen receptor-negative and/or patients with high Ki 67 levels (>20%) and HER2/neu-negative tumors were defined as luminal B. HER2/neu-positive patients were defined as HER2 type. Estrogen receptor-, progesterone receptor-, and HER2/neu-negative tumors were defined as triple negative. The histopathological parameters of the index side were recorded for BSBC, and the type of the surgery was recorded as mastectomy in the BSBC group if it was applied to any side. The local recurrence and metastatic status at the final follow-up were also recorded.

### Statistical analysis

The frequency and descriptive analyses of the cases were recorded. The qualitative data were analyzed using Fisher’s exact test, and the quantitative data were analyzed using the Mann-Whitney U test. Survival analyses were conducted using the Kaplan Meier test and the Cox multivariate regression test, and logistic regression tests were used for subgroup analyses. The data analysis was performed using SPSS software (SPSS Statistics for Windows, Version 17.0. Chicago: SPSS Inc).

## Results

The total number of patients included in the study was 507. There were 89 patients in the BSBC group, 271 patients in the MFBC group, and 147 patients in the MCBC group. Surprisingly, the MCBC patients were significantly older than the other two groups. The mean age in the MCBC group was 51.11 ± 11.9 years (range of 29 to 83 years). The mean ages of the MFBC and BSBC groups were 47.27 ± 11.63 years (range of 29 to 84 years) and 44.83 ± 9.67 years (range of 27 to 84 years), respectively (*P* <0.001). The majority of the MCBC patients were postmenopausal (*P* <0.001).

The BSBC cases had more foci than the MFBC and MCBC cases (mean focus number of 3.12 ± 1.46, 2.88 ± 1.54, and 2.3 ± 0.77, respectively; *P* <0.001). In addition, the mean tumor size was larger in the BSBC patients compared to that in the MFBC and MCBC patients (55.88 ± 23.7 mm, 22.09 ± 12.0 mm, and 34.96 ± 20.5 mm, respectively; *P* <0.001). The most common histological type in all groups was invasive ductal carcinoma (*P* = 0.001).

Compared to the other groups, the histological grade was higher in the MCBC group (*P* <0.001), and the majority of the patients were lymphatic vascular invasion-positive in the BSBC group (*P* <0.001).

There were no N0 patients in the BSBC group, and most of the N3 patients were in the BSBC group. This difference was statistically significant (*P* <0.001). A total of 13 N-positive patients did not undergo axillary surgery whilst their sentinel lymph node biopsy was positive; all 13 patients underwent additional axillary radiotherapy. No local recurrences occurred in these patients.

Estrogen receptor status and progesterone receptor status were also similar among the groups (*P* = 0.515 and *P* = 0.193, respectively). Compared to the other patient groups, there were more HER2/neu-positive tumors in the MCBC patient group (*P* = 0.006).

Whilst we cannot obtain the detailed treatment regimens we reported the obtainable treatment regimens for three groups (89 patients (100%) for the BSBC group, 230 patients (84.8%) for the MFBC group and 100 patients for the MCBC group). Due to the heterogeneity we did not apply statistical analyses.

The median follow-up time was 46.42 ± 23.2 months (range of 1 to 108 months). However, for each group the median follow-up was 49.63 ± 23.98, 44.29 ± 22.85, and 40.15 ± 19.92 months for MFBC, MCBC, and BSBC groups, respectively.

Local recurrence and mortality were not different among the groups (*P* = 0.263 and *P* = 0.996, respectively). Five local recurrences occurred in BSBC group, three of which were breast cancer recurrences and two of which were axillary cancer recurrences. A total of 14 local recurrences occurred in the MFBC group, eight of which were breast cancer recurrences and six of which were axillary cancer recurrences. Three local recurrences occurred in MCBC group, two of which were breast cancer recurrences and one of which was axillary cancer recurrence. Comparative analyses of these data are shown in Table 1.

**Table 1:** **Analyses of histopathological parameters and survival between the groups**

	BSBC	MFBC	MCBC	***P***
	(n = 89)	(n = 271)	(n = 147)	
Age	44.83 ± 9.67 (27–84) years	47.27 ± 11.63 (19–84) years	51.11 ± 11.94 (29–83) years	<0.001
Menopause Status				<0.001
Premenopausal	56 (62.9%)	168 (62.0%)	60 (40.8%)	
Postmenopausal	33 (37.1%)	103 (38.0%)	87 (59.2%)	
Number of tumors (İndex side for BSBC)	3.12 ± 1.46	2.88 ± 1.54	2.3 ± 0.77	<0.001
Tumor size (mm) (İndex side for BSBC)	55.88 ± 23.7	22.0 ± 12.0	34.9 ± 20.5	<0.001
Histological type				0.001
Invasive ductal	75 (84.2%)	198 (73%)	134 (91.1%)	
Mix	5 (5.6%)	23 (8.4%)	9 (6.1%)	
Invasive lobular	6 (6.7%)	33 (12.1%)	4 (2.7%)	
Others	3 (3.3%)	14 (6.1%)	0	
Histological Grade (Modified Bloom Richardson)				<0.001
I	10 (11.5%)	40 (15.0%)	34 (23.1%)	
II	66 (75.9%)	113 (42.3%)	36 (24.5%)	
III	11 (12.6%)	114 (42.7%)	77 (52.4%)	
Lymphatic vascular invasion				<0.001
Positive	68 (76.4%)	134 (49.4%)	77 (42.5%)	
Negative	21 (23.6%)	137 (50.6%)	70 (47.6%)	
Lymph node involvement				<0.001
N0	0	128 (47.2%)	68 (46.3%)	
N1	17 (19.1%)	83 (30.6%)	17 (11.6%)	
N2	41 (46.1%)	40 (14.8%)	34 (23.1%)	
N3	31 (34.8%)	20 (7.4%)	28 (19.0%)	
Estrogen receptor status				0.515
Positive	53 (59.6%)	147 (54.8%)	87 (59.2%)	
Negative	36 (40.4%)	124 (45.8%)	60 (40.8%)	
Progesterone receptor status				0.193
Positive	69 (77.5%)	195 (72.0%)	98 (66.7%)	
Negative	20 (22.5%)	76 (28.0%)	49 (33.3%)	
HER2/neu status				0.02
Positive	8 (9.0%)	16 (5.9%)	24 (14.2%)	
Negative	73 (90.1%)	255 (94.1%)	123 (83.7%)	
Molecular subtypes				0.03
Luminal A	46 (51.7%)	135 (49.82%)	69 (46.9%)	
Luminal B	16 (18.08%)	51 (18.8%)	19 (12.9%)	
Triple negative	19 (21.3%)	69 (25.5%)	35 (23.8%)	
HER2 type	8 (9.0%)	16 (5.9%)	24 (16.3%)	
Type of surgery				<0,001
BCS + SLN	0	132 (48.7%)	32 (21.8%)	
BCS + AD	23 (25.8%)	105 (38.7%)	4 (2.7%)	
MST + SLN	13 (14.6%)	11 (4.1%)	30 (20.4%)	
MRM	53 (59.6%)	23 (8.5%)	81 (55.1%)	
Adjuvant treatment modalities				Not applied
Chemotherapy	87 (97.7%)	141 (52.0%)	80 (54.4%)	
Radiotherapy	89 (100%)	225 (83.0%)	75 (51.0%)	
Endocrine therapy	60 (67.4%)	151 (55.7%)	85 (57.8%)	
Targeted therapy	8 (9.0%)	12 (4.4%)	21 (14.2%)	
Local recurrence	5 (5.6%)	14 (5.2%)	3 (2.0%)	0.263
Mortality	7 (7.9%)	22 (8.1%)	12 (8.2%)	0.996

AD: Axillary dissection; BCS: Breast conserving surgery; MRM: Modified radical mastectomy; MST: Mastectomy; SLN: Sentinel lymph node biopsy.

Local recurrence rates dependent on surgery type were analyzed (5.6% for MFBC, 2.0% for MCBC, and 5.2% for BSBC), and there was no significant difference between surgery types (95.0 CI of 98.5 to 104.25; *P* = 0.369). These low local recurrence rates could be explained by careful selection of patients for breast-conserving surgery. A trustable clean surgical margin was defined as at least 2 mm at all five centers, and re-excisions were made toward reaching these margins. Otherwise, a mastectomy was performed.

Univariate analyses of survival indicated significant differences in relation to histological type (*P* <0.001), histological grade (*P* = 0.024), estrogen receptor status (*P* <0.001), progesterone receptor status (*P* <0.001), lymph node involvement (*P* = 0.025), and molecular subtype (*P* <0.001). However, BSBC diagnosis, menopausal status, lymphatic-vascular invasion, and HER2/neu status were not statistically significant (*P* = 0.544, *P* = 0.092, *P* = 0.875, and *P* = 0.104, respectively). Having a mastectomy was associated with a poorer prognosis (*P* <0.001).

**Figure 1:**
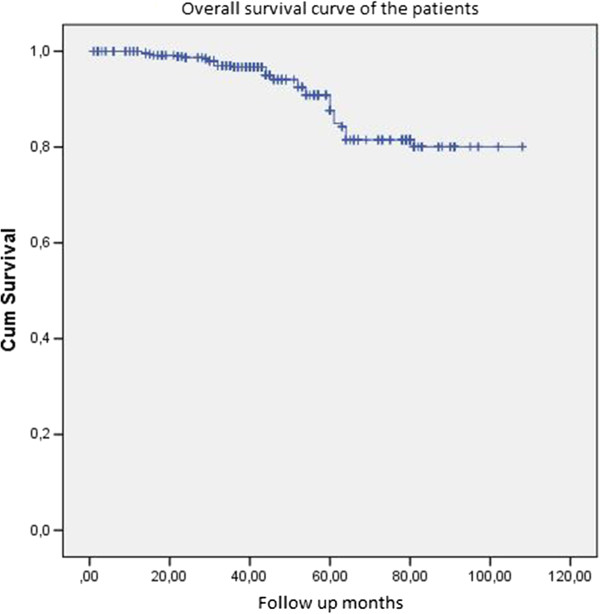
**Overall survival curve.**

**Figure 2:**
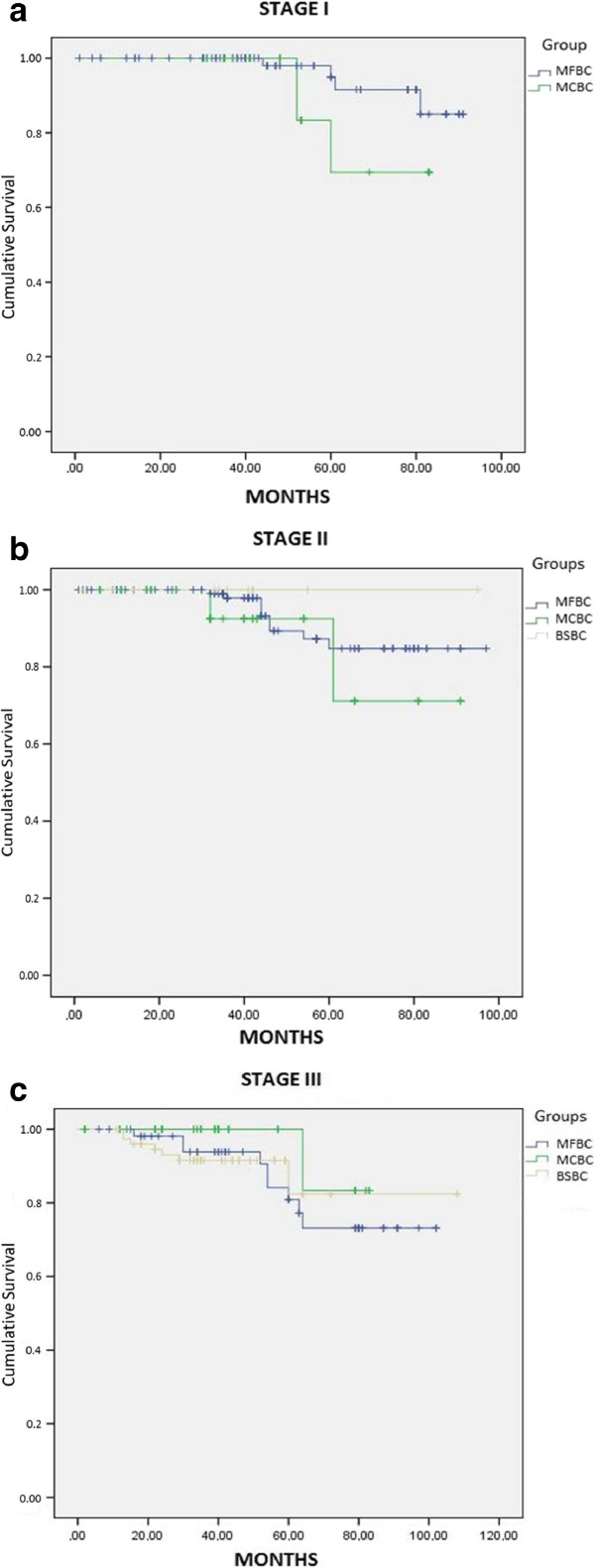
**Stage-dependent survival graphics of the groups. a**: Stage-dependent survival for Stage I patients. **b**: Stage-dependent survival for Stage II patients. **c**: Stage-dependent survival for Stage III patients.

There were not any statistically significant differences in overall survival rates (the five-year survival rate was 82.7%, 86.9%, and 90.5% for the BSBC, MFBC, and MCBC groups, respectively; *P* = 0.416) (Figure 1). The five-year disease-free survival rate was 82.7%, 80.7%, and 75.5% for the BSBC, MFBC, and MCBC groups, respectively (*P* = 0.024). When we compared the stage-dependent survival analyses of the three groups, there was no significant difference among the mortality rates (95.0 CI of 0.731 to 15.191, *P* = 0.87; 95.0 CI of 0.661 to 5.240, P = 0.73; and 95.0 CI of 0.106 to 1.452, *P* = 0.63 for Stage I, II, and III, respectively; Figure 2a, b, c).

Metastasis rates were different between the groups. The highest ratio was in the MFBC group (18.4%, 27 out of 271 patients) and the lowest ratio was in the BSBC group (9%, 8 out of 89 patients, *P* = 0.04). We believe that these ratios are the result of our small sampling size and that this caused a statistical bias regarding the metastatic ratios of this study.

We analyzed the usual prognostic parameters that were statistically significant in Kaplan-Meier analyses using the Cox multivariate regression test. Only lymph node status, lymphatic-vascular invasion, and estrogen receptor status had a statistically significant effect on survival (*P* = 0.006, *P* = 0.027, and *P* = 0.41, respectively) (Table 2). Progesterone receptor status alone did not have a statistically significant effect on survival. Table 3 shows the univariate and multivariate analyses data for the prognostic parameters.

**Table 2:** **Multivariate analyses for the effect of classical histopathological parameters on overall survival between the groups**

	***P***	95% CI	OR
Histological grade	0.027	0.198-0.905	4.913
Estrogen receptor status	0.41	0.116-0.954	4.190
Progesterone receptor status	0.171	-	-
Lymph node involvement	0.006	1.396-7.165	7.614
Molecular subtypes	<0.001	2.74-13.55	23.63

CI: Confidence Interval; OR: Odds Ratio.

**Table 3:** **Univariate analyses for the effect of classical histopathological parameters on overall survival between the groups**

	***P***
Overall survival between groups	0.544
Menopause status between groups	0.092
Histological type	0.488
Histological grade	0.024
Lymphovascular invasion	0.875
Estrogen receptor status	<0.001
P*rogesterone rece*p*tor status*	<0.001
HER2/neu status	0.104
Lymph node involvement	0.045
Molecular subtypes	<0.001

Finally, we analyzed the survival effect of molecular subtypes as luminal A, luminal B, triple negative, and HER2 type. While no survival difference was found between luminal A and B patients (*P* = 0.142), mortality in triple-negative patients was 5.11-fold greater than in luminal A patients (95.0% CI of 2.06 to 9.48, *P* <0.001; Table 3).

## Discussion

Multiple simultaneous tumor foci in the same breast are referred to as multifocal or multicentric, but there is no consensus on the terminology [13]. Classically, tumors in different quadrants are referred to as multicentric. Pathologists define multiple simultaneous primary lesions when there are two or more tumor foci without intervening malignant tissue [14]. Pathologists define tumors as multifocal when only one breast quadrant is involved, and they define tumors as multicentric when two or more quadrants are involved [14]. Radiologists do not have an exact definition, but tumors are usually considered multifocal when the distance between the tumors is less than or equal to 5 cm, and are considered multicentric when the distance between the tumors is more than 5 cm [15]. The estimated prevalence differs from 4 to 65%, and this variability is mainly due to the lack of standardization in the gross examination and sampling of breast specimens [16].

BSBC is still a subject of debate for oncology professionals. The data regarding BSBC are sophisticated, and the study results are confusing. We believe that the patient choice in the studies is the cause of this confusing status. Synchronous and metachronous tumors are all discussed similarly in these studies.

In addition, the comparisons of the groups are heterogeneous. Tumors that begin from more than one focus (such as BSBC) are compared with unilateral and unifocal tumors. At the same time, the biology of the tumors beginning from more than one focus should be different.

The literature reports are not all based on the same definition of BSBC [17]. A recent study evaluating 5292 patients in Germany found that differences in outcome between synchronous and metachronous cancers depend on time interval and that the optimal cut-off for the overall survival of BSBC is 4.5 months [18]. We believe the optimal definition of BSBC is cancers diagnosed simultaneously in both breasts or within three months (90 days) of diagnosis of the first tumor as described by Hartman *et al*. [4]. For these reasons, it is difficult to compare our results with the literature. We compared our results with recent literature in which results for BSBC were separately given for at least for 40 patients, and the definition of BSBC was similar to ours. Only a few studies have independently compared synchronous and metachronous breast cancers, and these studies have contradictory results [4, 19–21].

Roder *et al*. concluded that the risk for BSBC increases with age [21]. In general, other studies report a median age older than 50 years [22]. A significant portion of our BSBC patients were premenopausal (62.9%) with a median age that was younger (44.83 ± 9.67 years) than other Western studies, yet the median age was similar to that reported by Shi *et al*. (BSBC patient median age of 49 ± 15 years) [19]. Similarly, our MFBC and MCBC patients were younger (the median age for MFBC and MCBC was 47.27 ± 11.63 and 51.11 ± 11.94 years, respectively) than the ages reported in Western literature, but this result was similar to the ages reported in Eastern literature [23]. Recent literature evaluating 1492 consecutive breast cancer patients from Turkey has shown that 41.2% of the patients are younger than 50 years [24]. Furthermore, another study from Turkey evaluating MFBC and MCBC has reported that 51.4% of the patients are premenopausal [25]. These data suggest different biological behaviors of breast cancer in different races.

In our study, the mean tumor size was larger than reported in the literature for BSBC [19–22], yet all of these reported studies included patients with neo-adjuvant chemotherapy. Only one study from Italy excluded patients with neo-adjuvant chemotherapy, but the mean tumor size in our study was larger than that reported by Intra *et al.*[24].

The incidence of lobular histology is between 15 and 22% for BSBC in Western studies [20, 22, 26], but the incidence was 6.7% in our study, which was similar to the results reported by Eastern studies [23, 25]. A large study from British Columbia evaluating 25,320 breast cancer patients reported a lobular histology incidence of 13.6% [27]. The result for histological type was 12% in the study of Cabioglu *et al.*[28]. This percentage is slightly less than the ratio from British Columbia. Our cumulative ratio for MFBC and MCBC was 9.6%, and this ratio was slightly lower than both studies, but concordant with Eastern studies [23].

A substantial percentage of the tumors had high histological grades. This result was similar to the literature in which a histological grade of II or III was assigned to between 33.0 and 79.5% of BSBC patients and between 85.6 and 90.8% of MFBC and MCBC patients [20, 25]. However, no studies are available that compare histological grade in BSBC to that in MCBC and MFBC.

As confirmed by NCCN guidelines, a higher histological grade is an unfavorable factor for breast cancer. Thus, we analyzed the survival effect of histological grade by univariate and multivariate analyses. Histological grade had a negative effect on survival in both analyses (univariate analysis: *P* = 0.024; multivariate analysis: 95% CI of 0.198 to 0.905, *P* = 0.027).

The impact on the survival of these patients is a matter of discussion. Some publications conclude that BSBC patients have worse prognoses [20, 22, 26], and other reports do not refer to significant differences between unilateral and BSBC [26]. Indeed, the largest study of BSBC is a PhD thesis from Adelaide University (Australia) [20]. McCaul [20] analyzed 4424 women with BSBC who were diagnosed in the same month, and the results of his thesis suggest that tumor burden should be taken into consideration. He reported that bilateral stage I tumors have a similar prognosis when compared with unilateral stage I tumors, but that the survival of bilateral stage II tumors is worse than unilateral stage II tumors. However, a survival comparison of one side stage II and one side stage I tumors with unilateral stage II tumors showed that BSBC patients have a similar survival rate. In this study, we could not find a statistically significant difference in the five-year overall survival, but the five-year survival rates were lower for the BSBC group compared to the other groups. Perhaps a new classification in which the tumor burden of both breasts is included should be made.

Gene expression profiling has led to a new molecular classification of breast cancer with the triple-negative subgroup having the worse prognosis. As predicted, the triple-negative group had the worse prognosis in this study.

The ratio of HER2/neu positivity is smaller than the literature in the results of this study. We believe that the cause of this difference could be the small sampling size.

Thus imaging modalities become more important at the decision of BSBC-MFBC and MCBC, the role of magnetic resonance imaging (MRI) is another redundant point for the oncology specialists. Recent literature about the role of imaging modalities in the diagnosis of BSBC suggests that a family history of breast cancer, a multifocal breast tumor, or the presence of an invasive lobular carcinoma should be arguments for the realization of a breast MRI to eliminate contralateral malignancy [29]. Another prospective study from Turkey that explores the utility of positron emission tomography/computed tomography (PET/CT) scans to assess tumor multifocality and multicentricity suggests that: “However, the specificity of the MR is rather poor, being less than half that of PET/CT scans”. Thus PET/CT scan data should be considered before aggressive surgery is scheduled [30].

Only two case studies evaluating BSBC patients from Turkey have been published (with a total of 20 patients) [31, 32]. However, larger studies evaluating multifocal and multicentric breast cancers in Turkey are available [25, 28]. The number of patients reported in this study is the largest series in Turkey. It is important to report the status of BSBC patients in Turkey.

In conclusion, we need a common and accepted definition for BSBC. Prospective studies that include patients who fit this description should be conducted to determine the survival statistics for BSBC. Staging of BSBC should be investigated, and tumor burden should also be taken into account.

The data for treatment regimens were insufficient, which may affect the results of this study. In addition, the follow-up period was too short, and long-term results could be different. Thus, this study can only give a preliminary hypothesis regarding the short-term prognosis for tumors with multiple foci.

Of course the major limitation of this study is the fact that it is a retrospective analysis, as well as the known limitations and biases of the multicentric studies.

## Conclusions

In conclusion BSBC, MFBC, and MCBC had similar survival rates in this study. The multivariate analyses identified molecular subtypes and lymph node status as the main factors that affected survival. However, results from the literature are confusing because there are different definitions and different survival results. Thus, a decision about BSBC survival rates can be made only after a large trial with a standardized definition of BSBC is conducted.

## Abbreviations

AD: Axillary dissection
BS: bilateral synchronous
BSBC: Bilateral synchronous breast cancer
BC: breast cancers
BCS: Breast conserving surgery
CI: Confidence Interval
HG: histological grade
LVI: lymphovascular invasion
MST: Mastectomy
MRM: Modified radical mastectomy
MC: multicentric
MCBC: multicentric breast cancer
MF: multifocal
MFBC: multifocal breast cancer
NCCN: national comprehensive cancer network
OR: Odds Ratio.
